# Body Weight Deviations as Indicator for Resilience in Layer Chickens

**DOI:** 10.3389/fgene.2019.01216

**Published:** 2019-12-13

**Authors:** Tom V.L. Berghof, Henk Bovenhuis, Han A. Mulder

**Affiliations:** Wageningen University & Research Animal Breeding and Genomics, Wageningen, Netherlands

**Keywords:** resilience, immunity, disease resistance, natural antibody, production, body weight, deviation, micro-environment

## Abstract

Resilience is the capacity of an animal to be minimally affected by disturbances or to rapidly return to the state pertained before exposure to a disturbance. Less resilient animals are expected to be more susceptible to environmental perturbations, such as diseases, and will consequently show more and/or greater fluctuations in production than more resilient animals. Natural antibodies (NAb) are antibodies recognizing antigens without previous exposure to these, and are hypothesized to be an indication of general disease resistance. The objective of this research was to investigate genetic parameters of resilience indicators based on standardized body weight (BW) deviations and to investigate its relation with immunity (i.e. NAb) and disease resistance. Keyhole limpet hemocyanin-binding NAb were measured in layer chickens, which were selectively bred for high and low keyhole limpet hemocyanin-binding NAb levels during six generations. In addition, BW data of these layers were collected on a four-weekly interval from 4 weeks of age until 32 weeks of age. Standardized deviations of BW from an individual were compared to lines’ average BW (i.e. across individuals), and these were used to calculate resilience indicators: natural logarithm-transformed variance [ln(variance)], skewness, and lag-one autocorrelation of deviations (i.e. all within an individual). Heritabilities of resilience indicators were between 0.09 and 0.11. Genetic correlations between the three resilience indicators were between -0.20 and 0.40 (with high SE), which might suggest that the resilience indicators capture different aspects of resilience. Genetic correlations between resilience indicators and NAb were close to zero, which suggests that the resilience indicators and NAb capture different aspects of immunity. This might indicate that, in this dataset, environmental perturbations are only to a small extent affected by disease incidence, possibly due to a lack of disease occurrence. However, a lower estimated breeding value for ln(variance) was predictive for lower lesion scores after an avian pathogenic *Escherichia coli* inoculation and vice versa. In conclusion, this study shows that there is genetic variation in resilience indicators based on BW deviations in layer chickens, which opens up possibilities to improve resilience by means of selective breeding.

## Introduction

Resilience (or in other work referred to as robustness) is the capacity of an animal to be minimally affected by disturbances or to rapidly return to the state pertained before exposure to a disturbance (Colditz and Hine, 2016; Berghof et al., 2019b). Resilient livestock are essential for future livestock production as resilient livestock are easy-to-manage and require less individual treatment (Elgersma et al., 2018), which results in a greater production without negative effects on animals and farmers (Berghof et al., 2019b). Resilience can be of different nature based on the disturbance (e.g. disease, heat stress) (Colditz and Hine, 2016). Thus, “general” resilience is a composite trait consisting of different resilience types, such as disease-resilience and heat stress-resilience (Colditz and Hine, 2016; Friggens et al., 2017; Berghof et al., 2019b).

Improving general resilience on livestock animals can be achieved via different complementary strategies, of which one is breeding. In a recent paper, Berghof et al. (2019b) showed that economic values for resilience can be derived based on reduced labor and health costs. Scenarios for pig and dairy cattle breeding schemes showed a greater selection response when information on resilience was used in selection (Berghof et al., 2019b). These simulations suggest that resilience should be included in total merit indices of livestock. However, these simulations assumed the availability of suitable indicators for resilience, which is not the case in practice.

**Figure 1:**
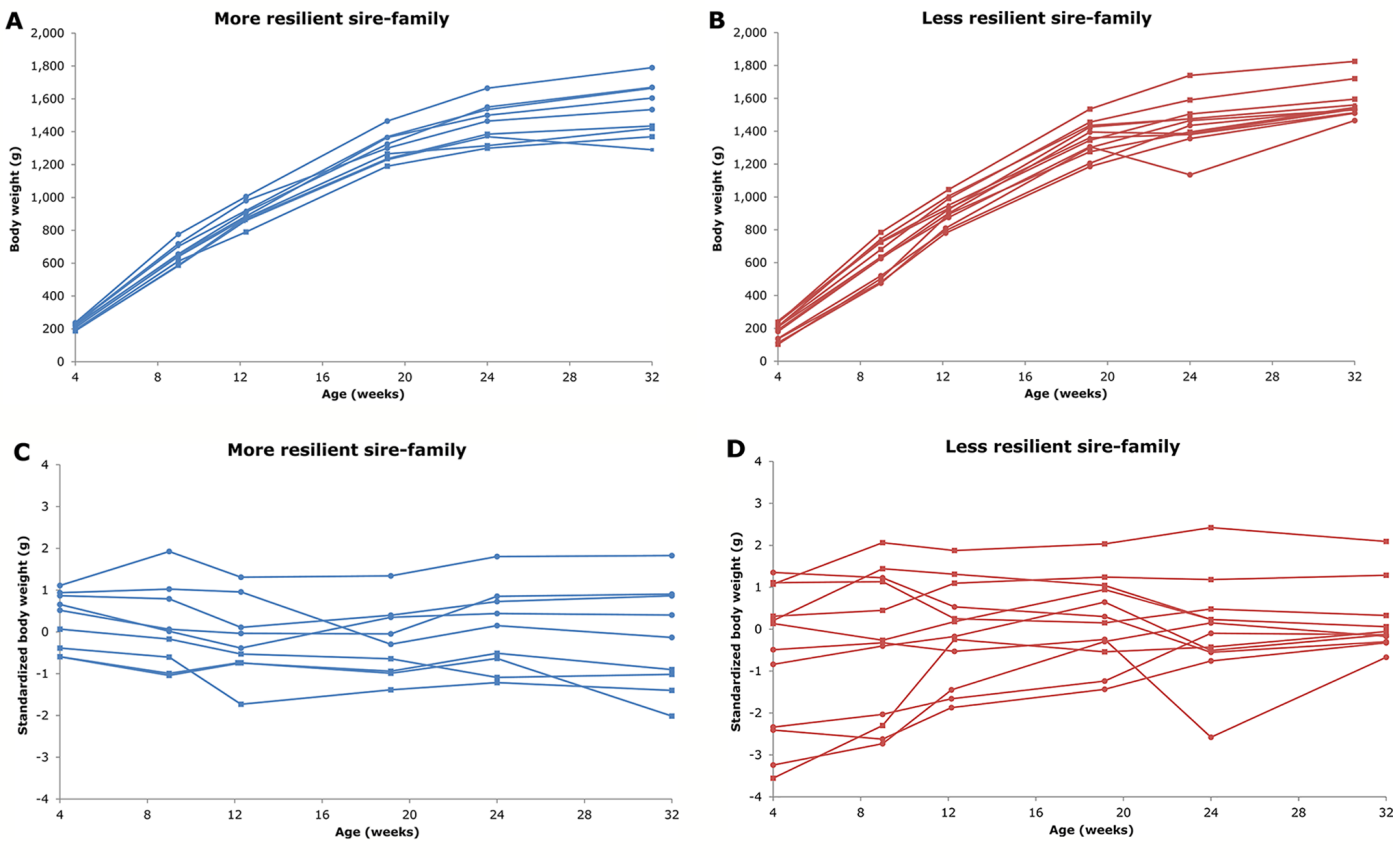
Examples of a more resilient sire-family (**A** and **C**; in blue) and a less resilient sire-family (**B** and **D**; in red) based on the sire's estimated breeding values for natural logarithm-transformed variance [ln(variance)]. **A** and **B** show body weights (BW) over time. **C** and **D** show standardized BW over time. **C** and **D** illustrate the difference in ln(variance) most easily: a more horizontal standardized BW line over time indicates a lower ln(variance) and thus a greater (hypothesized) resilience, and vice versa. The sire-families were obtained by mating the sire with two dams. The two families within the sire-family are indicated with different markers (round or square). Only BW of offspring with estimates for the resilience indicators (5 or more BW observations) are shown. The sire-families in this figure are families of generation 5 of the high line of the natural antibody (NAb)-selection experiment (i.e. the sires were generation 4).

Resilience indicators for individual animals have been proposed based on longitudinal data by calculating deviations of observed from expected production (Knap, 2009; Doeschl-Wilson et al., 2012; Colditz and Hine, 2016; Friggens et al., 2017). Deviations and their patterns in production traits have been shown before as indicative for health-related traits, although these approaches use only relatively-large, short-time deviations (e.g. De Haas et al., 2004; Codrea et al., 2011; Fischer et al., 2018; Nguyen-Ba et al., 2019; Revilla et al., 2019; Byrd et al., 2019). Berghof et al. (2019b), however, used a different approach to define three resilience indicators based on all production deviations due to unknown disturbances during a production cycle using concepts to study resilience of ecosystems (Scheffer et al., 2009; Scheffer et al., 2015; Scheffer et al., 2018): natural logarithm-transformed variance [ln(variance)] of deviations, skewness of deviations, and autocorrelation of deviations (Berghof et al., 2019b). These resilience indicators were proposed to have potential, because they are easy to obtain in livestock production data, cover the whole production period, and are expected to represent different aspects of resilience (Berghof et al., 2019b). More resilient animals are expected to show few(er) and smaller deviations compared to less resilient animals, because they are less influenced by disturbances. This is assumed to be independent of the nature and degree of the disturbances (i.e. no re-ranking), which are often unknown and never completely constant anyway. **Figure 1** illustrates a more resilient sire-family and a less resilient sire-family based on the sire's estimated breeding values (EBV) for ln(variance) of body weight (BW) deviations. More resilient animals are expected to have a smaller ln(variance) (i.e. closer to zero), and a skewness and an autocorrelation around zero compared to the population average (Berghof et al., 2019b).

Three studies have investigated the genetic potential of the variance of deviations of longitudinal data as an indicator for resilience in livestock and found favorable relations with health: Elgersma et al. (2018) and Poppe et al. (2020) found that fluctuations in daily milk yield in dairy cows are heritable, and are favorably and negatively genetically correlated to health and longevity traits (Elgersma et al., 2018; Poppe et al., 2020). Putz et al. (2019) found that fluctuations in daily feed intake and daily duration at the feeder in pigs are heritable, and favorably and negatively genetically correlated to mortality and treatment rate (Putz et al., 2019). In addition, many studies found heritable variation in uniformity in livestock species, which is the same as genetic heterogeneity of residual variance (see Hill and Mulder, 2010 and Elgersma et al., 2018 for overviews; e.g. Rowe et al., 2006; Mulder et al., 2007; Mulder et al., 2008; Ibáñez-Escriche et al., 2008a; Ibáñez-Escriche et al., 2008b; Mulder et al., 2009; Wolc et al., 2009; Neves et al., 2011; Wolc et al., 2012; Janhunen et al., 2012; Sae-Lim et al., 2015; Mulder et al., 2016; Sae-Lim et al., 2016; Sae-Lim et al., 2017). Thus, variance of production traits is heritable and seems to indicate resilience, however skewness and autocorrelation of production traits have not previously been investigated as potential indicators for resilience in livestock.

Natural antibodies (NAb) are antigen binding antibodies present in individuals without a (known) previous exposure to this antigen (Baumgarth et al., 2005). Previous studies showed that commercial layer chickens with high NAb levels binding keyhole limpet hemocyanin (KLH) were associated with lower mortality (Star et al., 2007; Sun et al., 2011; Wondmeneh et al., 2015). A NAb-selection experiment divergently selected layer chickens on total keyhole limpet hemocyanin (KLH)-binding NAb levels (IgTotal), resulting in a line with high NAb levels (High line) and a line with low NAb levels (low line) (Berghof, 2018). The High line showed increased antibody response to some antigens (Berghof et al., 2018a). Moreover, the High line had a 3.0 times reduced risk to die during an avian pathogenic *Escherichia coli* (APEC)-inoculation compared to chickens of the low line. Surviving High line chickens also had lower total lesion scores (i.e. morbidity) compared to surviving low line chickens at the end of the experiment (Berghof et al., 2019a). It was hypothesized that the NAb-selection lines differ in general (bacterial) disease resistance (Berghof, 2018). We hypothesize that the NAb-selection lines also differ in disease resilience and consequentially differ in general resilience (i.e. the resilience indicators).

The objectives of this study were:

1. to investigate the genetic parameters of resilience indicators based on four-weekly standardized BW deviations of layer chickens divergently selected for total KLH-binding NAb levels;
2. to investigate the relationship between resilience indicators and immunity by estimating the (genetic) correlation between resilience indicators and NAb levels; and
3. to investigate the relationship between resilience indicators and disease resistance by investigating the predictive ability of resilience indicators on APEC-resistance.

Given the current developments in the field of sensor technology, it is expected that large amounts of longitudinal data (“big data”) will become available for investigation of resilience based on deviations. This study explores the potential of resilience indicators based on longitudinal data. Moreover, this study is, as far as we know, the first to investigate genetic parameters of skewness and autocorrelation, and the first to investigate a direct relationship between resilience, immunity and disease resistance.

## Materials and Methods

### Study Populations

**Figure 2:**
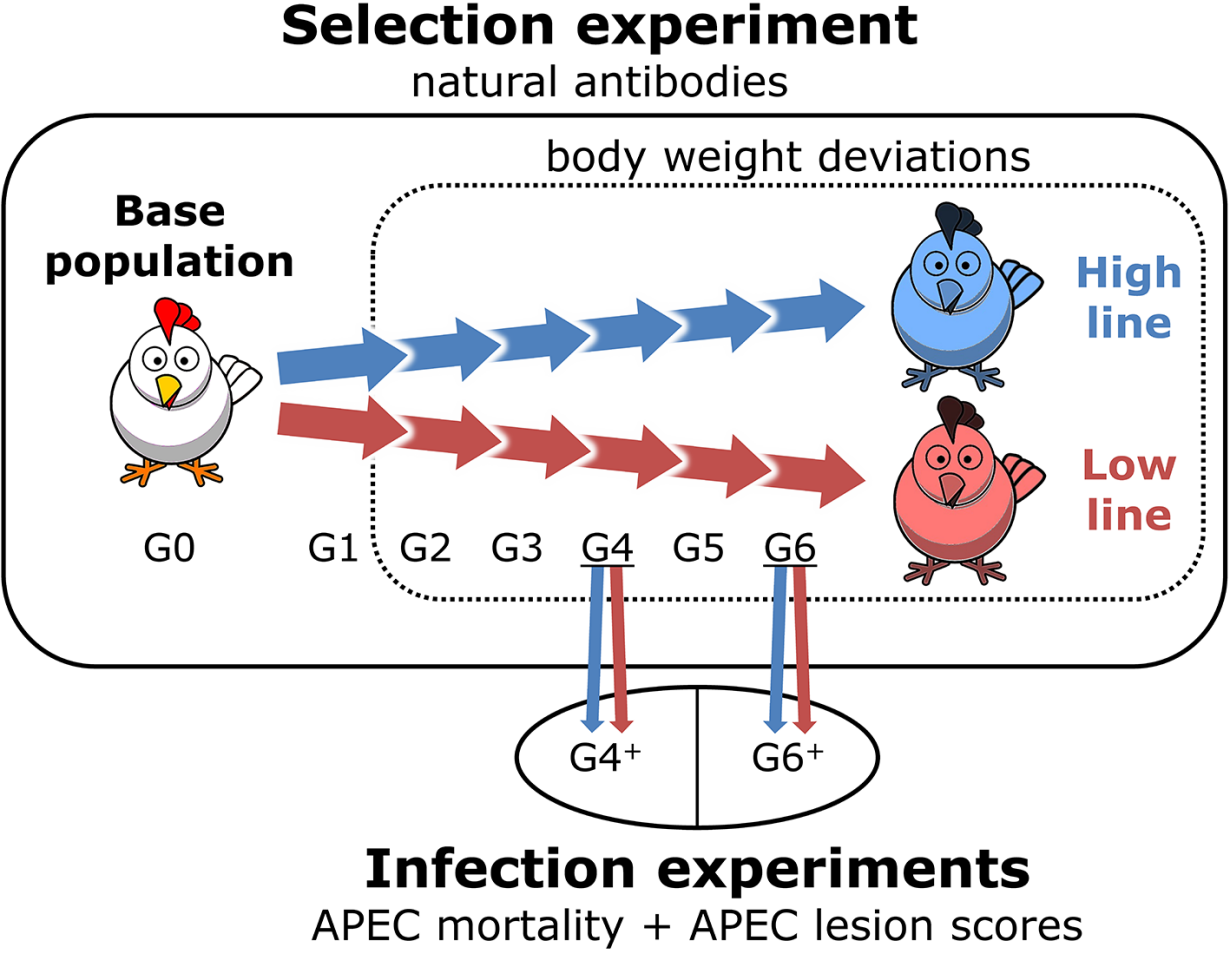
Overview of the study populations. Body weight deviations and natural antibody (NAb) were measured during the selection experiment. Avian pathogenic *Escherichia coli* (APEC) mortality and APEC lesion scores were measured during the infection experiments. Base population is the unselected population (G0) and the NAb-selection lines are generation (G)1 to G6. High line and Low line are the selected lines for high and low total keyhole limpet hemocyanin-binding NAb titers at 16 weeks of age, respectively. G4^+^ and G6^+^ are the additionally bred chickens for the infection experiments and are similar to G4 and G6 of the selection experiment.

The study populations consisted of the high and low NAb-selection lines in the selection experiment (including base population) and the NAb-selection lines in the infection experiments. **Figure 2** gives an overview of the study populations.

#### Selection Experiment

For objective 1 and objective 2, genetic parameters of resilience indicators based on BW deviations and their relations with NAb were investigated based on data of the NAb-selection experiment. The selection experiment-population consisted of an unselected (i.e. not for NAb selected) base population [generation (G)0] and six generations of NAb-selection lines [generation 1–6 (G1–G6)]. A steady increase in phenotypic differences in NAb and NAb EBV was observed over six generations between the high and low NAb-selection line, see Berghof (2018).

Briefly, the selection process was performed as follows:

- The G0 consisted of almost 3,700 purebred White Leghorn chickens (approximately 2,400 females and 1,300 males) from the “WA” line of Hendrix Genetics. Plasma samples of the studied chicken population were collected at 15 weeks of age (males) or 19 weeks of age (females). Selection of the breeding candidates was based on total KLH-binding NAb (IgTotal) titer (see below). For the high and low line, 25 males and 50 females with the highest and lowest titers were selected to breed G1. Each male was mated to 2 females and each female was mated to 1 male (adjusted from Berghof et al., 2018a).
- Incubation of eggs and housing of chickens for the selection lines (from G1 onwards) was at research facility “Carus” from Wageningen University & Research according to standard production practices. Each generation consisted of approximately 600 chickens per line. Plasma samples were collected at 16 weeks of age. Selection of the breeding candidates was based on total KLH-binding NAb (IgTotal) titer (see below).Within a line, the 25 best males and the 50 best females were selected to breed the next generation. Selected males and females were group housed from 0 to 16 weeks of age and individually housed from 16 weeks of age to 32 weeks of age. Non-selected males were culled after 16 weeks of age and non-selected females were group-housed from 0 weeks of age to 32 weeks of age. Chickens were not exchanged between lines. Each male was mated to 2 unrelated females (i.e. not mated with siblings or half-siblings) and each female was mated to 1 unrelated male (i.e. not mated with sibling or half-sibling) (adjusted from Berghof et al., 2018a).

More information about G0 can be found in Van Der Klein et al. (2015), and more information about the NAb-selection experiment can be found in Berghof et al. (2018a).

#### Infection Experiments

For objective 3, data of infection experiments of the NAb-selection lines were used to investigate the relationship between resilience and disease resistance. An additional G4 and G6 were bred during the NAb-selection experiment, and inoculated with APEC, see *Disease Resistance: APEC Mortality and APEC Lesion Scores* and Berghof et al. (2019a) for more details.

#### Resilience Indicators Based on Body Weight Deviations

BW were collected approximately every 4 weeks from 4 weeks of age to approximately 32 weeks of age for hens in the selection experiment, except at 28 weeks of age. Roosters were not included in the dataset, because only roosters selected for breeding the next generation of the selection experiment were kept until approximately 32 weeks of age.

BW records were visually checked for reliability across individuals of their respective cohort of line*generation*weighing moment with the Interactive Data Analysis tool from SAS^®^ software (SAS software V9.3 TS Level 1M2): 4 BW observations of different individuals were considered to be unreliable and were removed from the dataset. For these individuals, BW doubled between time point 1 and time point 2 and was subsequently reduced with more than 25% between time point 2 and time point 3. Time point 2 was then considered to be an unreliable BW observation and was removed.

BW were standardized to an average of zero and a standard deviation of one across individuals of their respective cohort of line*generation*weighing moment to correct for scaling effects during life and known cohort differences in BW (Berghof, 2018) (see **Supplementary Table 1** for descriptive statistics of the cohorts). As a consequence, the standardized BW records are equal to standardized deviations. Thus, deviations were calculated as:

$$deviation_{yz}=standardized\;BW_{yz}=\frac{BW_{yz}-{\bar{BW}}_{z}}{s_{z}},$$

where *BW_yz_* is BW *y* of the *z* ^th^ cohort, $\bar{BW_{z}}$ is the average BW of the *z* ^th^ cohort, and *s_z_* is the standard deviation of BW of the *z* ^th^ cohort.

The resilience indicators used in this study are the ln-transformed variance of deviations within an individual, the skewness of deviations within an individual, and the lag-one autocorrelation of deviations within an individual (Berghof et al., 2019b). The variance was ln-transformed with a natural logarithm (ln), because this is the commonly used scale to express the variance (also known as uniformity) in other studies and allows direct comparison of the additive genetic variance between studies (Hill and Mulder, 2010; Sell-Kubiak et al., 2015). The resilience indicators were calculated for each individual with 5 or more BW observations, which resulted in the removal of all individuals in G0 and G1 and removal of G2–G6 individuals without sufficient observations. The dataset contained 4,966 individuals before editing. A total of 3,393 individuals were removed (G0: 2,392 individuals, G1: 458 individuals, and G2–G6: 543 individuals). The final dataset for ln(variance) and skewness consisted of 1,593 individuals. Autocorrelation was only kept in the dataset if an individual had five or more subsequent observations in order to obtain at least 4 pairs of data points to estimate autocorrelation. This resulted in an additional removal of 110 individuals, which resulted in 1,463 individuals in the final dataset for autocorrelation.

The ln(variance) of deviations of the *j* ^th^ individual was calculated as:

$$ln(variance_{j})=ln\left(\frac{\Sigma_{i=1}^{n_{j}}{\left(x_{ij}-{\bar{x}}_{j}\right)}^{2}}{n_{j}-1}\right),$$

where *x_ij_* is deviation *i* of the *j* ^th^ individual, ${\bar{x}}_{j}$ is the mean of deviations of the *j* ^th^ individual, and *n_j_* is the number of deviation observations of the *j* ^th^ individual.

The skewness of deviations of the *j* ^th^ individual was calculated as:

$$skewness_{j}=\frac{n_{j}}{\left(n_{j}-1)(n_{j}-2\right)}\sum_{i=1}^{n_{j}}{\left(\frac{x_{ij}-{\bar{x}}_{j}}{\sqrt{s_{j}^{2}}}\right)}^{3},$$

where *n_j_* is the number of deviation observations of the *j* ^th^ individual, *x_ij_* is deviation *i* of the *j* ^th^ individual, ${\bar{x}}_{j}$ is the mean of deviations of the *j* ^th^ individual, and $s_{j}^{2}$ is the variance of deviations of the *j* ^th^ individual being calculated as: $s_{j}^{2}=\frac{\Sigma_{i=1}^{n_{j}}{\left(x_{ij}-{\bar{x}}_{j}\right)}^{z}}{n_{j}-1}$, where *x_ij_* is deviation *i* of the *j* ^th^ individual, ${\bar{x}}_{j}$ is the mean of deviations of the *j* ^th^ individual, and *n_j_* is the number of deviation observations of the *j* ^th^ individual.

The autocorrelation of deviations of the *j* ^th^ individual was calculated as:

$$autocorrelation_{j}=\frac{\Sigma_{i=1}^{n_{j}-1}(x_{ij}-{\bar{x}}_{j})(x_{(i+1)j}-{\bar{x}}_{j})}{\Sigma_{i=1}^{n_{j}}{\left(x_{ij}-{\bar{x}}_{j}\right)}^{2}},$$

where *n_j_* is the number of pairs of subsequent deviations of the *j* ^th^ individual, *x_ij_* is deviation *i* of the *j* ^th^ individual, ${\bar{x}}_{j}$ is the mean of deviations of the *j* ^th^ individual, and *x*_(_ *_i_*_+1)_ *_j_* is the subsequent deviation of deviation *i* of the *j* ^th^ individual.

**Table 1** shows an overview of the number of observations for the three resilience indicators per line and generation.

**Table 1:** Average, standard deviation (in parentheses) and number of observations (in *italic*) for the three resilience indicators and keyhole-limpet hemocyanin-binding natural antibody (NAb) titers at 16 weeks of age per generation per line (H, high NAb-selection line; L, low NAb-selection line) and total.

Trait	Generation													Total
	0^a^	1		2		3		4		5		6		
		H	L	H	L	H	L	H	L	H	L	H	L	
ln(variance)	–	–		-1.27	-1.20	-1.56	-1.31	-1.25	-1.20	-1.34	-1.11	-1.51	-1.57	-1.32
				(0.88)	(0.92)	(1.04)	(1.00)	(0.85)	(0.80)	(1.01)	(0.87)	(0.98)	(1.06)	(0.94)
				*117*	*139*	*108*	*95*	*209*	*224*	*160*	*186*	*177*	*158*	*1,573*
skewness	–	–		-0.29	-0.18	-0.08	-0.27	-0.13	-0.16	-0.15	-0.21	-0.18	-0.11	-0.17
				(0.82)	(0.91)	(0.95)	(1.03)	(0.84)	(0.89)	(0.82)	(0.88)	(0.84)	(0.80)	(0.87)
				*117*	*139*	*108*	*95*	*209*	*224*	*160*	*186*	*177*	*158*	*1,573*
autocorrelation	–	–		0.23	0.25	0.29	0.17	0.27	0.25	0.22	0.23	0.39	0.39	0.27
				(0.42)	(0.45)	(0.56)	(0.57)	(0.39)	(0.40)	(0.52)	(0.56)	(0.48)	(0.55)	(0.48)
				*115*	*135*	*94*	*82*	*209*	*224*	*148*	*168*	*157*	*131*	*1,463*
IgTotal NAb^bc^	7.3	6.4	5.9	6.4	5.2	8.1	6.8	6.4	4.3	6.6	4.6	7.5	5.5	-^c^
	(1.4)	(1.2)	(1.3)	(1.3)	(1.4)	(1.6)	(1.8)	(1.3)	(1.6)	(1.3)	(1.7)	(0.9)	(1.4)	
	*3,664*	*467*	*479*	*385*	*435*	*264*	*262*	*460*	*455*	*578*	*557*	*372*	*335*	*8,713*
IgM NAb^c^	7.5	6.6	6.1	6.7	5.7	6.4	5.2	6.1	4.3	6.7	4.0	6.3	3.6	-^c^
	(1.3)	(1.0)	(1.0)	(0.8)	(0.9)	(0.8)	(1.0)	(0.9)	(1.1)	(0.9)	(1.2)	(1.1)	(1.2)	
	*3,664*	*467*	*479*	*385*	*435*	*264*	*262*	*460*	*455*	*578*	*557*	*372*	*335*	*8,713*
IgG NAb^c^	6.3	6.3	5.8	6.0	5.2	5.8	5.0	6.0	4.4	6.5	4.8	6.7	4.4	-^c^
	(1.6)	(1.3)	(1.4)	(1.3)	(1.4)	(1.5)	(1.8)	(1.4)	(1.6)	(1.4)	(1.7)	(1.6)	(1.9)	
	*3,664*	*467*	*479*	*385*	*435*	*264*	*262*	*460*	*455*	*578*	*557*	*372*	*335*	*8,713*

a Base population, not selected for NAb.

b Selection criterion.

c NAb are measured on a relative scale and comparison over generations is therefore not possible.

### Immunity: Natural Antibodies

NAb optical densities (OD) were determined in individual plasma samples by an indirect two-step ELISA and calculated as described by Berghof et al. (2018c).

Briefly, plasma samples were 1:10 pre-diluted with dilution buffer. Flat-bottomed, 96-well medium binding plates were coated with 2 µg/ml KLH in 100 µl coating buffer and incubated at 4°C overnight. After washing for 6 s with tap water containing Tween^®^ 20, plates were tapped dry. The 1:10 pre-dilution of the samples were further diluted with dilution buffer to 1:40, 1:160, 1:640, and 1:2,560 test dilutions. Duplicate standard positive plasma samples were stepwise 1:1 diluted with dilution buffer. The plates were incubated for 1.5 h at room temperature (20–25°C). After washing, plates were incubated with 1:20,000-diluted anti-chicken IgG heavy and light chain (IgTotal) labeled with horse radish peroxidase (PO), or 1:20,000-diluted anti-chicken IgM labeled with PO, or 1:40,000-diluted anti-chicken IgG(Fc) labeled with PO, and incubated for 1.5 h at room temperature (20–25°C). After washing, binding of the antibodies to KLH was visualized by adding 100 µl substrate buffer at room temperature (20–25°C). After 15 min the reaction was stopped with 50 µl of 1.25 M H_2_SO_4_. OD were measured at 450 nm [adjusted from Berghof et al. (2018c)].

Antibody titers were calculated as described by Berghof et al. (2018c) (based on Frankena, 1987). Briefly, the OD of the duplicate standard positive plasma samples were averaged for each plate. Logit values of the OD per plate were calculated, and a linear regression line of the logit OD against the respective log_2_-dilution values of the averaged duplicate standard positive plasma samples was fitted. Titers of the plasma samples per plate were calculated using the linear regression line (adjusted from Berghof et al., 2018c). **Table 1** shows an overview of the number of observations for NAb titers per line and generation.

### Disease Resistance: Avian Pathogenic *Escherichia coli* Mortality and Avian Pathogenic *Escherichia coli* Lesion Scores

Two inoculation experiments were performed with additionally bred G4 and G6 chickens, as described by Berghof et al. (2019a). Care was taken to select one male and one female of each family (where possible) per treatment to have a balanced representation of both NAb-selection lines.

Briefly, at 8 days of age, chickens of both selection lines received an intratracheal inoculation of 0.2 ml phosphate buffered saline containing 10^8.20^ colony-forming units (CFU)/milliliter APEC in the additional G4 (197 individuals in total), or 10^6.64^ CFU/ml APEC in the additional G6 (180 individuals in total) (adjusted from Berghof et al., 2019a). These treatments were selected, because they had the largest variation in mortality (10^8.20^ CFU/ml) and lesion scores (10^6.64^ CFU/ml) (Berghof et al., 2019a, see Berghof et al., 2019a for all treatments).

After inoculation, chickens were checked every 2 h for the first 4 days post inoculation, and subsequently every 8 h until the end of the experiment (approximately 170 h post inoculation). Mortality was scored as the percentage of chickens alive at a certain moment divided by the total number of chickens at the start of the experiment. At 15 days of age (7 days post inoculation), all surviving chickens were euthanized [adjusted from Berghof et al. (2019a)].

Lesion scores (i.e. morbidity) were macroscopically assessed on the surviving chickens at the end of the experiment based on Van Eck and Goren (1991). Lesion scoring was performed on the left thoracic air sac, the right thoracic air sac, the pericardium, and the serosal surface of the liver, always in the same order. Severity of the lesion scores were defined as follows: 0: no lesions, 0.5: one single pinhead-sized inflammatory spot, 1: two or more pinhead-sized spots, 2: fibrinous patches on various locations, and 3: extensive fibrinous patches (Van Eck and Goren, 1991). The total lesion score is the sum of scores for the four individual locations [adjusted from Berghof et al. (2019a)].

### Statistical Analyses

#### Genetic Parameters of Resilience Indicators

Genetic parameters for the resilience indicators based on four-weekly BW deviations of layer chickens were estimated.

The following linear animal model was used for estimating variance components for the resilience indicators:

$$y_{abcj}=\mu +nObs_{a}+Gen_{b}+Sel_{c}+{\left(Gen*Sel\right)}_{bc}+a_{j}+e_{abcj},$$

where *y_abcj_* is the resilience indicator ln(variance), skewness, or autocorrelation, *µ* is the overall mean, *nObs_a_* is the fixed effect of the number of observations for the resilience indicator (*a* = 5–7 for ln(variance) and skewness, and *a* = 4–6 for autocorrelation), *Gen_b_* is the fixed effect of generation (*b* = 2–6), *Sel_c_* is the fixed effect of individual housing (after 16 weeks of age) of the females selected for producing the next generation within the selection experiment (with *c* being not selected or being selected), (Gen *Sel)*_bc_* is the fixed effect of the interaction term between *Gen_b_* and *Sel_c_*, *a_j_* is the random additive genetic effect of the *j* ^th^ animal assumed to be $∼N(0,\;A\sigma_{a}^{2})$, *e_abcj_* is the residual term assumed to be $∼N(0,\;I\sigma_{e}^{2})$. Assumed (co)variance structures of the random model terms are $A\sigma_{a}^{2}$ and $I\sigma_{e}^{2}$, in which **A** is the additive genetic relationship matrix based on the pedigree consisting of 11,360 individuals from in total 13 generations, $\sigma_{a}^{2}$ is the additive genetic variance, **I** is an identity matrix, and $\sigma_{e}^{2}$ is the residual variance.

Heritabilities were calculated as

$$h^{2}=\frac{\sigma_{a}^{2}}{\sigma_{p}^{2}},$$

where $\sigma_{a}^{2}$ is the additive genetic variance, and $\sigma_{p}^{2}$ is the phenotypic variance being calculated as: $\sigma_{p}^{2}=\sigma_{a}^{2}+\sigma_{e}^{2}$, where $\sigma_{a}^{2}$ is the additive genetic variance and $\sigma_{e}^{2}$ is the residual variance. The likelihood ratio test was used to test whether estimated heritabilities were significantly different from zero, comparing the tested model to a model in which the additive genetic variance was fixed at a value of 0.000001. The likelihood ratio test was -2ln(Λ(x)) with $\Lambda (x)=\frac{\max[L_{0}|x]}{\max[L_{1}|x]}$, where L_0_ is the likelihood under the null hypothesis with the additive genetic variance fixed at 0.000001, L_1_ is the likelihood under the alternative hypothesis without variance components constrained, and x is the given dataset. Significance was assessed with the likelihood-ratio test assuming that the likelihood ratio follows a $\chi_{1}^{2}$-distribution.

The genetic coefficient of variation (GCV) was calculated as:

$$GCV=\frac{\sqrt{\sigma_{a}^{2}}}{\mu },$$

where $\sigma_{a}^{2}$ is the additive genetic variance of the resilience indicator skewness or autocorrelation, and *µ* is the overall mean of the resilience indicator. For ln(variance), the GCV is calculated as $\sqrt{\sigma_{a}^{2}}$, because the ln-transformation implicitly assumes an exponential model. Therefore $\sqrt{\sigma_{a}^{2}}$ is without units and division by *µ* is redundant (Mulder et al., 2007; Hill and Mulder, 2010).

To estimate the similarity between resilience indicators, phenotypic and genetic correlations between resilience indicators were estimated based on bivariate analyses using the linear animal models for the resilience indicators described above.

#### Resilience Indicators and Immunity

To investigate the relationship between resilience indicators and immunity, the phenotypic and genetic correlations between resilience indicators and NAb, and the correlated selection response of resilience indicators in the NAb-selection lines were estimated.

Phenotypic and genetic correlations between resilience indicators and NAb were estimated based on bivariate analyses using the linear animal models for the resilience indicators described above and the following linear animal model for NAb as described by Berghof (2018):

$$y_{djk}=\mu +Plate_{d}+a_{j}+dam_{k}+e_{djk}$$

where *y_djk_* is the IgTotal, IgM, or IgG titer, *µ* is the overall mean, *Plate_d_* is the fixed effect of plate *d* on which a sample was analyzed (*d* = 1-422), *a_j_* is the random additive genetic effect of the j^th^ animal assumed to be $∼N(0,\;A\sigma_{a}^{2})$, *dam_k_* is the random effect of the k^th^ dam assumed to be $∼N(0,\;I\sigma_{m}^{2})$, and *e_djk_* is the residual term assumed to be $∼N(0,\;I\sigma_{e}^{2})$. Assumed (co)variance structures of the random model terms are $A\sigma_{a}^{2}$, $I\sigma_{m}^{2}$, and $I\sigma_{e}^{2}$, in which **A** is the additive genetic relationship matrix based on the pedigree consisting of 11,360 individuals from in total 13 generations, $\sigma_{a}^{2}$ is the additive genetic variance, **I** is an identity matrix, $\sigma_{m}^{2}$ is the maternal variance, and $\sigma_{e}^{2}$ is the residual variance. Note that the plate effect accounts for confounded effects on the samples, such as sex, storage, and analysis effects.

The correlated selection response of resilience indicators was investigated by plotting the average EBV of the resilience indicators (obtained from the univariate linear animal models) for each line and generation.

#### Resilience Indicators and Disease Resistance

To investigate the relationship between resilience indicators and disease resistance, the predictive ability of the resilience indicators on APEC mortality and APEC lesion scores was investigated.

EBV of the resilience indicators and IgTotal (obtained from the univariate linear animal models) were used as predictors for mortality in infection experiment 1 (G4) and total lesion scores in infection experiment 2 (G6). Because individuals in the challenge experiments were not part of the NAb-selection lines (i.e. additional G4 and G6), the EBV basically represent parents' EBV averages.

The statistical model used for performing the survival analysis to investigate the relationship between resilience indicators and APEC mortality in the additional G4 was a Cox proportional hazards model (Cox, 1972; Kleinbaum and Klein, 2012):

$$h_{j}(t)=h_{0}(t)\times exp[X\beta ],$$

where *h_j_*(*t*) is a hazard function describing the probability at time *t* (*t* = 0–170 h post inoculation) for death to occur on the *j* ^th^ APEC-inoculated chicken (*j* = 1–197), *h*_0_(*t*) is an unspecified baseline hazard function at time *t*, *exp* is the natural exponential function (i.e. *e^x^* with ***x*** being ***Xβ***), ***X*** is a design matrix containing predictors with ***β*** being the vector with parameters. The investigated predictors were $EBV_{ln{\left(variance\right)}_{j}}$, $EBV_{skewness_{j}}$, or $EBV_{autocorrelation_{j}}$ for the *j* ^th^ APEC-inoculated chicken. To account for the effect of selection on IgTotal NAb, the model was also extended with $EBV_{IgTotal_{j}}$ for the *j* ^th^ APEC-inoculated chicken.

Conditional probabilities were calculated for chickens that died at the same moment (i.e. ties), according to Kalbfleisch and Prentice (2011). Chickens that were euthanized at the end of the experiment were censored (adjusted from Berghof et al., 2019a).

The statistical model used for performing the analysis to investigate the relationship between resilience indicators and APEC lesion scores in the additional G6 was an analysis of covariance (i.e. a general linear model):

$$y_{j}=\mu +X\beta +e_{j},$$

where *y_j_* is the total lesion score on the *j* ^th^ surviving APEC-inoculated chicken (*j* = 1-180), *µ* is the overall mean, ***X*** is a design matrix containing predictors with ***β*** being the vector with parameters, and *e_j_* is the residual term assumed to be $∼N(0,\;I\sigma_{e}^{2})$ where **I** is an identity matrix and $\sigma_{e}^{2}$ is the residual variance. The investigated predictors were $EBV_{ln{\left(variance\right)}_{j}}$, $EBV_{skewness_{j}}$, or $EBV_{autocorrelation_{j}}$ for the *j* ^th^ APEC-inoculated chicken. To account for the effect of selection on IgTotal NAb, the model was also extended with $EBV_{IgTotal_{j}}$ for the *j* ^th^ APEC-inoculated chicken.

#### Miscellaneous

Genetic parameters of NAb have already been reported based on (part of) this data elsewhere, and will therefore not be reported here (Berghof et al., 2015; Berghof, 2018).

Statistical analyses were performed using ASReml 4.1 (Gilmour et al., 2014) for “Genetic Parameters of Resilience Indicators” and “Resilience Indicators and Immunity,” and by using SAS^®^ software (SAS software V9.3 TS Level 1M2) for “Resilience Indicators and Disease Resistance.”

Significance was declared for p-values ≤ 0.05 and tendency to significance was declared for p-values ≤ 0.10.

## Results

### Genetic Parameters of Resilience Indicators

Variance components, heritabilities, and GCV of the resilience indicators ln(variance), skewness, and autocorrelation are reported in **Table 2**. The additive genetic variance ($\sigma_{a}^{2}$) for ln(variance) was 0.09, for skewness $\sigma_{a}^{2}$ was 0.07, and for the autocorrelation $\sigma_{a}^{2}$ was 0.02. Heritabilities were estimated to be 0.10 for ln(variance) and 0.09 for skewness, and were significantly different from zero. However, the heritability for autocorrelation was estimated to be 0.11 and tended to significance (p = 0.053). GCV was 0.30 for ln(variance), 1.56 for skewness, and 0.52 for autocorrelation. Note that the means of skewness and autocorrelation were close to zero, which may inflate their GCV. Thus, resilience indicators based on BW deviations are heritable and show high genetic variability.

**Table 2:** Variance components and SE (in parentheses), heritability and SE (in parentheses), and the genetic coefficient of variation (GCV) of the three resilience indicators {i.e. natural logarithm-transformed variance [ln(variance)], skewness, and autocorrelation}.

	ln(variance)	Skewness	Autocorrelation
$\sigma_{a}^{2}$	0.09 (0.03)	0.07 (0.03)	0.02 (0.01)
$\sigma_{e}^{2}$	0.78 (0.04)	0.70 (0.03)	0.21 (0.01)
$\sigma_{p}^{2}$	0.87 (0.03)	0.77 (0.03)	0.23 (0.01)
Heritability	0.10 (0.04)	0.09 (0.04)	0.11 (0.04)^a^
GCV	0.30	1.56	0.52

a Not significantly different from zero; p-value = 0.053. Significance was assessed with the likelihood-ratio test assuming that the likelihood ratio follows a $\chi_{1}^{2}$-distribution.

Phenotypic and genetic correlations among resilience indicators are reported in **Table 3**. Phenotypic correlations (r_p_) among resilience indicators were all weak (-0.10 ≤ r_p_ ≤ 0.32). The genetic correlations (r_g_) between the resilience indicators were low to moderate: the genetic correlation was -0.20 between ln(variance) and skewness, 0.40 between ln(variance) and autocorrelation, and 0.07 between skewness and autocorrelation, all with high SE. Thus, the weak correlations suggest that the resilience indicators capture different aspects of BW deviations.

**Table 3:** Phenotypic (above diagonal) and genetic correlations (below diagonal) and SE (in parentheses) of the three resilience indicators from the linear animal model {natural logarithm-transformed variance [ln(variance)], skewness, and autocorrelation}.

	ln(variance)	Skewness	Autocorrelation
**ln(variance)**	–	-0.10 (0.03)	0.32 (0.03)
**skewness**	-0.20 (0.28)	–	0.04 (0.03)
**autocorrelation**	0.40 (0.24)	0.07 (0.29)	–

### Resilience Indicators and Immunity

Phenotypic and genetic correlations of the resilience indicators and IgTotal, IgM, and IgG NAb are reported in **Table 4**. The phenotypic and genetic correlations between the resilience indicators and NAb were low: -0.03 ≤ r_p_ ≤ 0.02 and -0.09 ≤ r_g_ ≤ 0.08, all with high SE. Thus, the resilience indicators are genetically different from NAb, i.e. they contain hardly any common genetic variation.

**Table 4:** Phenotypic and genetic correlations and SE (in parentheses) of the three resilience indicators {natural logarithm-transformed variance [ln(variance)], skewness, and autocorrelation} and the natural antibodies (NAb).

	Phenotypic correlations			Genetic correlations		
	IgTotal	IgM	IgG	IgTotal	IgM	IgG
**ln(variance)**	-0.03 (0.03)	-0.01 (0.03)	-0.03 (0.03)	-0.09 (0.12)	-0.09 (0.12)	-0.06 (0.13)
**skewness**	-0.02 (0.03)	0.02 (0.03)	-0.02 (0.03)	0.01 (0.12)	-0.01 (0.13)	0.01 (0.13)
**autocorrelation**	-0.03 (0.03)	0.02 (0.03)	-0.02 (0.03)	0.02 (0.12)	0.08 (0.12)	0.01 (0.13)

**Figure 3:**
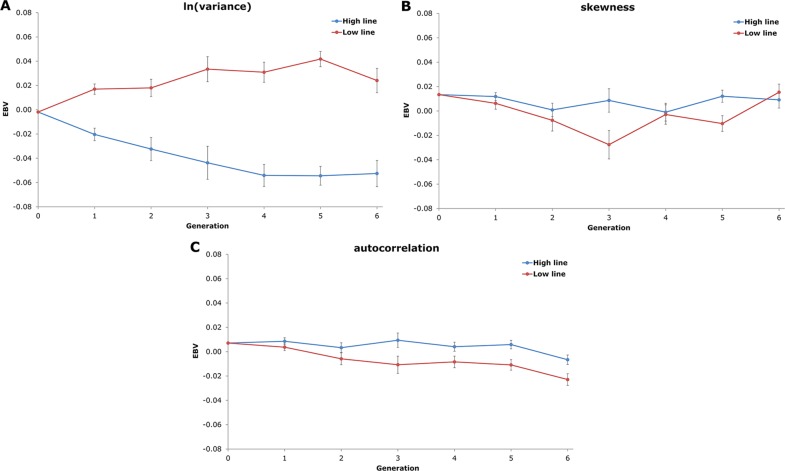
Average estimated breeding values (EBV) and standard errors of the two natural antibody (NAb)-selection lines (High line in blue and Low line in red) over six generations of selection for the three resilience indicators from the linear animal model (**A**: natural logarithm-transformed variance; **B**: skewness; and **C**: autocorrelation). Only EBV of females are shown. EBV of generation 0 and generation 1 are based on offspring phenotypes (i.e. no own phenotypes).

Average EBV of the two NAb-selection lines for the three resilience indicators are shown in **Figure 3**. In agreement with the estimated genetic correlation, the High line showed a negative trend in average EBV_ln(variance)_ and the low line showed a positive trend. EBV_skewness_ and EBV_autocorrelation_ averages did not show clear line differences and remained around zero. Thus, even though the genetic correlations between resilience indicators and NAb were low, EBV of ln(variance) showed a small correlated selection response for IgTotal NAb.

### Resilience Indicators and Disease Resistance

The predictive ability of EBV of the resilience indicators and IgTotal NAb for APEC mortality is shown in **Table 5**. EBV_IgTotal_ significantly predicted mortality (hazard ratio = 0.59; p = 0.01). None of the EBV of the resilience indicators did significantly predicted mortality, neither as single predictor, nor in combination with EBV_IgTotal_. Thus, the resilience indicators do not predict APEC-induced mortality.

**Table 5:** Predictors for avian pathogenic *Escherichia coli* mortality.

Predictor	# Predictors	HR	p
EBV_NAb_	1	0.59	0.01
EBV_ln(variance)_	1	1.98	0.63
EBV_skewness_	1	2.60	0.59
EBV_autocorrelation_	1	33.70	0.26
EBV_NAb_	2	0.54	0.01
+ EBV_ln(variance)_		0.26	0.39
EBV_NAb_	2	0.59	0.01
+ EBV_skewness_		1.89	0.70
EBV_NAb_	2	0.58	0.01
+ EBV_autocorrelation_		39.90	0.22

Predictors are estimated breeding values (EBV) for natural antibodies (NAb) (EBV_NAb_) and EBV for resilience indicators (natural logarithm-transformed variance [ln(variance)], skewness, and autocorrelation) (EBV_resilience indicator_). Predictors were tested as one covariate (i.e. EBV_NAb_ or EBV_resilience indicator_) and two covariates (i.e. EBV_NAb_ and EBV_resilience indicator_). Shown is the hazard ratio (HR) and the p-value of the HR.

The predictive ability of EBV of the resilience indicator and IgTotal NAb for APEC morbidity is shown in **Table 6**. EBV_IgTotal_ tended to significantly predict mortality (β = -0.50; p = 0.09). EBV_ln(variance)_ significantly predicted lesion scores (p = 0.03). The regression coefficient was positive (β = 6.20) and therefore favorable: greater EBV_ln(variance)_ (i.e. lower resilience) was associated with greater lesion scores (i.e. larger disease impact). The EBV_ln(variance)_ difference (minimum = -0.33, maximum = 0.19) accounted for a maximal total lesion score difference of 3.2 (out of 12), while EBV_IgTotal_ difference (minimum = -1.45, maximum = 1.41) accounted for a maximal total lesion score difference of 1.4. EBV_ln(variance)_ was significant when solely included as a predictor in the model, but only tended to significance (p = 0.07) when EBV_IgTotal_ was included. This suggests that EBV_ln(variance)_ and EBV_IgTotal_ partly compete to explain the same variance by the model. EBV_skewness_ and EBV_autocorrelation_ did not significantly predict lesion scores. Thus, EBV of the resilience indicator ln(variance) had a favorable relationship to APEC-induced lesion scores.

**Table 6:** Predictors for avian pathogenic *Escherichia coli* total lesion scores.

Predictor	# Predictors	β	p
EBV_NAb_	1	-0.50	0.09
EBV_ln(variance)_	1	6.20	0.03
EBV_skewness_	1	5.85	0.20
EBV_autocorrelation_	1	-5.70	0.76
EBV_NAb_	2	-0.37	0.23
+ EBV_ln(variance)_		5.36	0.07
EBV_NAb_	2	-0.38	0.07
+ EBV_skewness_		4.89	0.14
EBV_NAb_	2	-0.54	0.09
+ EBV_autocorrelation_		-6.49	0.60

Predictors are estimated breeding values (EBV) for natural antibodies (NAb) (EBV_NAb_) and EBV for resilience indicators (natural logarithm-transformed variance [ln(variance)], skewness, and autocorrelation) (EBV_resilience indicator_). Predictors were tested as one covariate (i.e. EBV_NAb_ or EBV_resilience indicator_) and two covariates (i.e. EBV_NAb_ and EBV_resilience indicator_). The total lesion score is the sum of scores (0-3) for the left thoracic air sac, the right thoracic air sac, the pericardium, and the serosal surface of the liver. Shown is the regression coefficient β and the p-value of the regression coefficient.

## Discussion

Breeding for improved resilience seems a promising strategy to obtain trouble-free livestock which is easy-to-manage, and has a greater welfare and health. Berghof et al. (2019b) proposed three resilience indicators to measure resilience based on deviations between expected production and observed production: ln(variance) of deviations, skewness of deviations, and autocorrelation of deviations (Berghof et al., 2019b). Recent studies suggest that fluctuations of longitudinally observed production traits might indeed be related to improved (disease) resilience in livestock (Elgersma et al., 2018); Putz et al., 2019; Poppe et al., 2020). Alternative resilience indicators like skewness and autocorrelation, however, have not been reported before (but is investigated in dairy cattle by Poppe et al., 2020). Breeding for greater NAb levels has been shown to improve APEC-resistance in chickens (Berghof et al., 2019a) and greater NAb levels have been hypothesized to improve or to be indicative for improved general disease resistance (Berghof, 2018). This study is the first to investigate heritabilities of ln(variance), skewness, and autocorrelation of longitudinal-observed deviations, and to relate these resilience indicators with immunity and disease resistance. The obtained results increase our understanding of the biological mechanisms underlying resilience in animals.

### Genetic Parameters of Resilience Indicators

The additive genetic variance of ln(variance) was within the range of previously reported estimates based on variation of different traits in different livestock species (e.g. Rönnegård et al., 2013; Vandenplas et al., 2013; Sell-Kubiak et al., 2015; Mulder et al., 2016; Iung et al., 2017; Elgersma et al., 2018), including BW variation in chickens (Rowe et al., 2006; Mulder et al., 2009; Wolc et al., 2009). Although reported heritabilities for ln(variance) are mostly low (0.00–0.10), GVC are generally considerable (0.15–0.30), which was observed for ln(variance) in this study as well. This indicates that there are good prospects for the genetic improvement of ln(variance) and thus for genetic improvement of resilience.

This study is the first study to investigate skewness and autocorrelation of deviations. Heritability estimates of skewness and autocorrelation were similar to the heritability of ln(variance). Poppe et al. (2020) estimated a heritability for autocorrelation based on daily milk deviations similar to this study, although the GCV was considerably lower (0.07–0.17) due to a much lower additive genetic variance. In contrast to our study, they estimated a very low heritability of 0.01 for skewness based on daily milk deviations and a low GCV (0.05–0.10) due to a much lower additive genetic variance compared to this study (factor 10 lower). Three major differences between Poppe et al.'s study and this study might underlie these differences observed for skewness and autocorrelation: 1. Poppe et al. used daily observations with a minimum of 50 observations per individual in contrast to this study's four-weekly observations with a minimum of five observations per individual. Poppe et al.'s approach captures (smaller, shorter) disturbances more accurately and is therefore expected to be a more accurate representation of resilience (as discussed in Berghof et al., 2019b); 2. Poppe et al. used milk yield deviations as an indicator. Milk yield is known to respond fast to environmental factors (i.e. disturbances) and changes in milk yield can be observed between days. In contrast, BW responds slowly to environmental factors, except for enteric diseases, and, generally, changes in BW only become apparent after several days. Thus, daily milk yield deviations can be a “fast resilience indicator,” while BW deviations (even if taken daily) are a “slow” resilience indicator. Therefore, it is expected that resilience indicators based on daily milk yield deviations are different from resilient indicators based on BW deviations; and 3. Poppe et al. based the expected production of an individual on the individual's lactation curve in contrast to this study's cohort average (line*generation*weighing moment). To approximate this method, deviations based on Gompertz curves for growth of each individual were used to estimate resilience indicators (results not shown). However, due to the small number of BW observations, deviations were almost completely absorbed into the Gompertz curve parameters, which resulted in very small resilience indicators. Therefore, fitting the Gompertz curve or fitting any other growth curve seems not appropriate when the number of observations is small, which effectively results in a low number of degrees of freedom. On the contrary, when using deviations from cohort averages to calculate resilience indicators, genetic differences in individual growth curves might end up as deviations from the cohort averages. As a consequence, resilience and growth curves are confounded. To investigate this potential confounding, the genetic correlations between the resilience indicators and the Gompertz curve's parameter a (estimated BW after the growth phase), parameter b (estimated BW before the growth phase), and parameter c (the estimated growth during the growth phase) were estimated (results not shown). The correlations were around zero between the resilience indicators and parameters a and b, which indicates that the resilience indicators are independent of the maximum BW and the initial BW. This is expected, because all resilience indicators are (mathematically) independent of averages. However, from the definitions of the three parameters, it is most likely that parameter c would yield non-zero genetic correlations with the resilience indicators. Indeed, the estimated genetic correlation between skewness and parameter c was -0.58 (SE = 0.17), while the genetic correlations between parameter c and ln(variance), and between parameter c and autocorrelations were close to zero. This suggests that skewness based on cohort averages captures partly individual differences in growth rate, and this might explain the differences observed for skewness between the study of Poppe et al. (2020), who used individual lactation curves, and this study. This finding does not per se exclude skewness based on BW deviations as a resilience indicator, because the individual growth differences might be a consequence of differences in resilience. It can be concluded that using cohort averages to calculate deviations is useful when the number of observations is low, but skewness of deviations based on cohort averages may also capture individual genetic differences in trait curves.

Genetic correlations between resilience indicators were low. This might indicate that some of the resilience indicators are not predictive or that the different resilience indicators capture different aspects of resilience (as argued above). However, it cannot be excluded that the genetic correlations are actually greater than estimated in this study and consequently that the resilience indicators are more alike, because of the large SE of the estimations. The large SE might be a consequence of the low frequency and impact of (disease) disturbances in the study population's environment. However, similar results were observed in the study of Poppe et al. (2020) under commercial settings, supporting the hypothesis of a different genetic makeup and different information of the resilience indicators (Berghof et al., 2019b), even though these indicators are based on the same deviations. In order to genetically improve resilience, the different resilience indicators need to be combined in a selection index, if such an index indeed predicts resilience better than any of the individual indicators alone.

In previous studies, ln(variance) was investigated by using double-hierarchical generalized linear models (DHGLM), which accounts for the mean and variance of deviations simultaneously (Rönnegård et al., 2010; Felleki et al., 2012; Mulder et al., 2013). DHGLM analyses are computationally and mathematically more challenging to perform and to achieve convergence, which makes implementations of these models into breeding programs also practically challenging. Nevertheless, a DHGLM was used to analyze the BW data and results were compared to the simpler approach reported here (results not shown). Interestingly, the additive genetic variance of the DHGLM-ln(variance) was equal to the additive genetic variance of ln(variance) with a high genetic correlation (0.995). This shows that the more complex DHGLM approach, though theoretically more accurate, can be approximated very well by a more simplistic approach (similar to Sell-Kubiak et al., 2015), which can be more easily implemented in practice.

Possible factors influencing the BW deviations are permanent environmental effects and maternal environmental effects. Permanent environmental effects exist on BW, because of the repeated measurements. However, we condensed the BW information to one estimate of a resilience indicator per animal, and therefore permanent environmental effects are not identifiable. Maternal environmental effects could not be estimated (results not shown), because for most of the dams too few (i.e. <5) offspring were present in the dataset. Not accounting for maternal effects might result in overestimation of the genetic variance. Genetic analyses of BW at different time points did have significant maternal environmental effects at all time points (Berghof et al., in preparation). When maternal environmental effects were estimated for the resilience indicators, a significant maternal environmental effect was found for autocorrelation. However, this also completely absorbed the genetic variance (i.e. heritability was not significantly different from zero), confirming difficulties to disentangle genetic variance and maternal variance with a low number of offspring per dam in this dataset. Nevertheless, this indicates that environmental effects may influence resilience indicators and should be considered in future studies whenever possible.

The proposed resilience indicators by Berghof et al. (2019b) are defined based on longitudinal data on a commonly-measured production trait. This study used 5 to 7 BW measurements for estimating the heritability of ln(variance). The ln(variance) heritability in this study was clearly greater than ln(variance) heritability estimates based on one observation per individual (e.g. Janhunen et al., 2012; Sae-Lim et al., 2015; Iung et al., 2017; see also Hill and Mulder, 2010 for a review). This fits within the proposed idea of high-frequency BW measurements (Friedman et al., 2012; Revilla et al., 2019) and the strong increase in heritability estimates with the addition of a few observations for an individual (**Figure 2** from Berghof et al., 2019b). Moreover, the current technological developments are expected to allow more frequent (i.e. daily) measurements on different phenotypes in all livestock species in the near future, including chickens (Friggens et al., 2017; Mulder, 2017; Berghof et al., 2019b). This study is therefore also an exploratory study to investigate the potential of different resilience indicators based on longitudinal data.

### Resilience Indicators and Immunity

To investigate the relationship between resilience and immunity, the genetic correlations between the three resilience indicators and NAb were investigated in chickens divergently selected for NAb levels (see Berghof, 2018 for more information). High levels of KLH-binding NAb were previously associated with lower mortality, improved immunity, and increased disease resistance in chickens (Lammers et al., 2004; Star et al., 2007; Sun et al., 2011; Wondmeneh et al., 2015; Berghof, 2018; Berghof et al., 2018a; Berghof et al., 2019a). Thus, NAb are expected to influence both the resistance (i.e. “minimally affected”) and the recovery (i.e. “rapidly return”), which are both intrinsic parts of (the definition of) resilience (Berghof et al., 2019b). We hypothesized that chickens selectively bred for greater NAb levels have a greater resilience compared to chickens selectively bred for lower Nab levels.

Genetic correlations between the resilience indicators and NAb were low, and thus, in contrast to our hypothesis, shared common mechanisms seem not to be present. This means that the resilience indicators do not account for disease resistance, or, more likely, do not cover disease resistance in this study population due to a too low level of disease pressure to exploit genetic differences under the standardized conditions of the research facility. It is well-known that in high challenge environment more genetic variation in resistance or resilience is observed compared to “normal” environments (Drangsholt et al., 2011; LaFrentz et al., 2016; Putz et al., 2019). For example, Putz et al. (2019) investigated resilience indicators in pigs housed in a “natural disease challenge model” (i.e. an environment with a high multifactorial disease pressure). They found relatively high heritabilities for variation (0.15–0.26), and favorable correlations between variation and mortality, and variation and number of treatments (0.37–0.85) (Putz et al., 2019). In the same study population, NAb (measured before entering the high challenge environment) were predictive for fewer medical treatments and a greater resilience (i.e. decreased day-to-day fluctuations in feed intake) (Tibbs et al., 2018). Although the phenotypic correlations were weak (< |0.09|; Tibbs et al., 2018), they are similar to the phenotypic correlations in this study. We are currently investigating the relation between resilience indicators and NAb in cross-bred populations housed in conventional production system. This will give more insight in their relationship under more challenging conditions. Anyhow, we hypothesize for now that NAb are informative for disease resilience before disturbances (i.e. diseases) are present, while the resilience indicators are informative for disease resilience after disturbances.

Remarkably, in preliminary work on the resilience indicators in animals up to G5, larger favorable genetic correlations (approximately -0.3) were found between ln(variance) and NAb (Berghof et al., 2018b). The preliminary study (G0–G5) and this study (G0–G6) have similar variance components, which suggests that G6 is similar to G0–G5. However, reported SE of both studies are large (around 0.2) due to a small study population, and consequently the difference in genetic correlations might be due to sampling. In addition, the discrepancy between G0–G5 and G0–G6 can also indicate that G6 added different information to the dataset regarding the relationship between NAb and resilience. G6 did have two notable differences compared to the other generations: 1. an *E. coli* infection was present in the flock during the first two weeks of life and the animals were treated with antibiotics. It can be expected that such an event would actually increase BW variation. However, that seems not the case. Alternatively, NAb levels and NAb level development during early life might have been influenced by this event; and 2. G6 was bred from parents of approximately 50 weeks of age due to other experimental work, while parents of the other generations were between 30 and 35 weeks of age. Such an age difference can result in for example differences of maternal antibody transfer to offspring, which can permanently influence humoral immunity (Klipper et al., 2004; Lammers et al., 2004; Gharaibeh et al., 2008; Hasselquist and Nilsson, 2009; Ulmer-Franco et al., 2012). However, maternal effects on NAb levels did not seem to be different in G6 (results not shown). Nevertheless, this remains speculative and future studies will have to give more insight in the genetic relationship between resilience and NAb, and the influence of maternal effects (e.g. dam age).

### Resilience Indicators and Disease Resistance

As stated above, a possible lack of disease challenges in the study population can explain an apparent weak relationship between resilience indicators and NAb. Berghof et al. (2019a) performed two APEC-challenge experiments to measure disease resistance in the NAb-selection lines. Although the experiments are too short to measure BW deviations to obtain resilience indicators on the challenged individuals, EBV for resilience indicators for the challenged individuals were obtained from the genetic parameter estimation. The predictive ability of EBV of resilience indicators on APEC-induced mortality and total lesion scores in the infection experiments was investigated to obtain insights in the relationships between resilience indicators and the response to diseases, i.e. APEC.

EBV_skewness_ and EBV_autocorrelation_ were not predictive for mortality or lesion scores. This is in line with Poppe et al. (2020), who concluded that skewness and autocorrelation of deviations in milk yield seem less useful as resilience indicators in dairy cows. Therefore, their potential use as resilience indicators seems to be limited, but needs further investigation in other study populations.

EBV_ln(variance)_ was predictive for lesion scores, but not for mortality. Possibly, lesion scores represent the capacity to deal with infections (i.e. not dying) and thus might be closer to the definition of resilience (i.e. recovery). Chickens with lower EBV_ln(variance)_ (i.e. a greater resilience) had lower lesion scores compared to chickens with greater EBV_ln(variance)_ (i.e. a lower resilience). EBV_ln(variance)_ predicted up to 25% of the total lesion score, which was twice as much as the EBV for IgTotal NAb, i.e. the selection criterion. Compared to ln(variance), this difference might be due to: 1. the protective function of NAb being more related to disease resistance (and mortality) rather than recovery, which is circumvented in this experiment by applying a standardized dose of an infectious agent; 2. the immunological function of NAb being more related to the initiation of the (humoral) adaptive immune response, which is barely initiated within the experimental period (i.e. one week is too short) (Berghof et al., in preparation); or 3. APEC-resistance being not solely dependent on NAb levels. Nevertheless, some common background in resilience and immunity/disease resistance seems to be present, since EBV of ln(variance) and NAb accounted for the same model variance. Thus, in line with previous studies (Elgersma et al., 2018; Putz et al., 2019; Poppe et al., 2020), ln(variance) seems to have predictive capacity for disease resilience.

## Conclusion

This study described three proposed resilience indicators in chickens: ln(variance), skewness, and autocorrelation of standardized BW deviations. All three resilience indicators were heritable, and suggest to capture different parts of resilience. In contrast to our hypothesis, NAb (as a measure of immunity) were not or only weakly genetically correlated with resilience indicators in this study population. Thus, resilience indicators and NAb seem not to be under common genetic control, suggesting that diseases cause only a small (negligible?) part of day-to-day disturbances. However, this might be a consequence of a lack of disease challenges during life. In addition, hints of a small common genetic background between ln(variance) and NAb were found: 1. the high NAb-selection line had a greater resilience compared to the low NAb-selection line based on EBV of ln(variance); and 2. individuals with a lower EBV for ln(variance) had lower APEC-induced lesion scores. Thus, ln(variance) might be an indicator for disease resilience and can, together with NAb, be included in breeding indices to improve resilience and immunity.

This study is, as far as we know, the first to investigate genetic parameters of skewness and autocorrelation, and the first to investigate a direct relationship between resilience phenotypes (as defined by Berghof et al., 2019b) and the immune system. Overall, this study suggests that ln(variance) is a promising resilience indicator, because it shows a (weak) relationship to immunity and disease resistance in a relatively high hygiene environment. More studies are needed to investigate the potential for skewness and autocorrelation, but these parameters seem less promising. Anyhow, the genetic variation found for the proposed resilience indicators gives ample opportunity to genetically improve resilience in chickens.

## Data Availability Statement

Data are available upon request, after approval of both the authors and Hendrix Genetics. Contact the corresponding author by e-mail.

## Ethics Statement

Ethical review and approval was not required for the animal study because Collection of samples and data of the base population was done according to Hendrix Genetics protocols, under the supervision of Hendrix Genetics employees. Samples and data were collected as part of routine data collection in a commercial breeding program for layer chickens in The Netherlands. Samples and data were collected on a breeding nucleus of Hendrix Genetics for breeding purposes and are a non-experimental, agricultural practice, regulated by the Act Animals and the Royal Decree on Procedures. The Dutch Experiments on Animals Act does not apply to non-experimental, agricultural practices. An ethical review by the 'Dierexperimentencommissie’ (Animal Experiment Committee) was therefore not required. No extra discomfort was caused by sample collection for the purpose of this part of the study. The selection and infection experiments were approved by the ‘Dierexperimentencommissie’ (Animal Experiment Committee) of Wageningen University & Research according to Dutch law (Experiment Codes Selection experiment: 2012105, 2013091, 2014058, and 2014093; Experiment Codes Infection experiments: 2014126 and 2016057) and the ‘Centrale Commissie Dierproeven’ (Central Committee Animal Experiments) of the Dutch government (numbers Infection experiments: 201521, and 2015357). Written informed consent was obtained from the owners for the participation of their animals in this study.

## Author Contributions

TB, HB, and HM designed the research. TB performed the research, collected the data, analyzed the data, and wrote the manuscript. HM assisted with the genetic analyses and interpretation of results. HB and HM revised the manuscript. All authors approved the final version.

## Funding

This work is part of the research programme “Divergent selection for natural antibodies in poultry” with project number 12208, which is financially supported by the Netherlands Organisation for Scientific Research. This work is financially supported by Netherlands Organisation for Scientific Research Earth and Life Sciences (NWO-ALW; ALWSA.2016.4). This work is in kind supported by Hendrix Genetics.

## Conflict of Interest

This research was in kind supported by Hendrix Genetics. Except for this support, no other shared interests (e.g. employment, consultancy, patents, products) exist between Hendrix Genetics and the authors.

All authors declare that the research was conducted in the absence of any commercial or financial relationships that could be construed as a potential conflict of interest.

The reviewer AD-W declared a past co-authorship with one of the authors HM to the handling editor.
