# Supplementary material for: Body Weight Deviations as Indicator for Resilience in Layer Chickens
Source: Front Genet. 2019 Dec 13;10:1216. doi: 10.3389/fgene.2019.01216 (PMC6923720; doi:10.3389/fgene.2019.01216)
Supplement: Supplementary file 1 [file DataSheet_1.pdf]

Supplementary Material

Body Weight Deviations as Indicator for Resilience in Layer Chickens

Tom V.L. Berghof*, Henk Bovenhuis, Han A. Mulder

*** Correspondence:** T.V.L. Berghof: tom.berghof@wur.nl

1 Supplementary Table 1

Supplementary Table 1. Average, standard deviation (in parentheses) and number of (unstandardized) body weight (BW) observations (in *italic*) per weeks of age for each line*generation*weighing moment-cohort (H = high natural antibody-selection line, L = low natural antibody-selection line).

	**Generation**									
	**2**		**3**		**4**		**5**		**6**	
**Trait**	**H**	**L**	**H**	**L**	**H**	**L**	**H**	**L**	**H**	**L**
BW4	211	209	178	165	184	174	205	198	187	172
	(37)	(37)	(38)	(36)	(34)	(28)	(29)	(26)	(35)	(32)
	138	*164*	*131*	*123*	*216*	*230*	*298*	*314*	*184*	*175*
BW8	621	612	523	505	576	544	651	629	638	593
	(82)	(81)	(81)	(74)	(77)	(67)	(64)	(65)	(84)	(86)
	140	*158*	*114*	*105*	*221*	*231*	*298*	*315*	*181*	*179*
BW12-13	915	892	939	905	901	861	912	891	946	899
	(93)	(87)	(97)	(88)	(83)	(80)	(71)	(72)	(86)	(88)
	129	*155*	*115*	*105*	*215*	*230*	*297*	*315*	*178*	*168*
BW15-16	1,182	1,154	1,099	1,065	1,158	1,117	-		1,185	1,095
	(114)	(107)	(88)	(67)	(84)	(84)			(102)	(107)
	115	*139*	*110*	*95*	*214*	*229*			*168*	*159*
BW17-20	1,367	1,336	-		1,261	1,204	1,330	1,273	-	
	(112)	(116)			(121)	(120)	(101)	(102)		
	117	*139*			*210*	*225*	*289*	*309*		
BW21-24	1,505	1,473	1,339	1,286	1,405	1,350	1,447	1,410	1,529	1,487
	(110)	(91)	(103)	(92)	(120)	(110)	(121)	(122)	(111)	(122)
	118	*138*	*112*	*103*	*206*	*222*	*161*	*183*	*177*	*162*
BW32-35	1,483	1,458	1,579	1,479	1,499	1,429	1,552	1,555	1,528	1,494
	(147)	(118)	(153)	(127)	(138)	(136)	(130)	(120)	(119)	(130)
	116	*132*	*96*	*84*	*203*	*218*	*150*	*174*	*177*	*159*
